# Book Review

**DOI:** 10.1120/jacmp.v6i2.2132

**Published:** 2005-05-21

**Authors:** Timothy Schultheiss

**Affiliations:** ^1^ Radiation Physics Department City of Hope Medical Center 1500 East Duarte Road Duarte California 91010

## Abstract

*Radiation Therapy Physics*, by William R. Hendee, Geoffrey S. Ibbott, and Eric G. Hendee, 3rd ed., John Wiley & Sons, Inc., 2005; ISBN 0‐471‐39493‐9; list price $125

*Medical Radiation Physics* was first published in 1970, at a time when most physicists came to medical physics from another field. In 1981, when the first edition of *Radiation Therapy Physics* was published, the same was still true. As one of these physicists, I owe a debt to William Hendee for filling in gaps in both my formal and practical education in medical physics through the publication of these fine books.

Since the publication of these early editions, medical physics has changed in virtually every particular. Who we are, how we learn, and what we do are all different. The audience for these books was and still is the entire team involved in the radiation treatment of patients with cancer. The third edition of *Radiation Therapy Physics* seeks to address these changes. Reaching and teaching residents, physicists, engineers, dosimetrists, therapists, and nurses with a single text seems nearly an unachievable goal. Because of the variation in backgrounds that students in these professions bring to the classroom, the degree of satisfaction each finds in the third edition of *Radiation Therapy Physics* must also vary. Furthermore, with such a broad target audience, it is not possible to maintain a consistent intellectual level.

Because the second edition sold out too quickly for me to purchase a copy, I cannot compare the second and third editions. Although I do own the first edition, the time elapsed between the third and the first edition is too great to allow for a fair comparison between the two books.

This third edition is divided into 16 logically sequenced chapters:

1. Atomic Structure and Radioactive Decay
2. Production of X Rays
3. Interaction of X Rays and Gamma Rays
4. Radiation Units
5. Measurement of Ionizing Radiation
6. Calibration of Megavoltage Beams of X Rays and Electrons
7. Dosimetry of Radiation Units
8. Treatment Planning by Manual Methods
9. Diagnostic Imaging and Application to Radiation Therapy
10. Computer Systems
11. Computer‐based Treatment Planning
12. Sources for Implant Therapy
13. Brachytherapy Treatment Planning
14. Radiation Protection
15. Quality Assurance
16. Advances in Radiation Therapy

Each chapter begins with Objectives, contains numerous figures, examples, and marginal commentary, and ends with a Summary. There is an average of about 10 problems at the end of every chapter, except the last. Extensive use is made of marginal comments. This has the effect of changing the feel of the text. Some may find the separate numbering of figures versus margin figures somewhat confusing and the marginal comments themselves distracting. However, others may enjoy the diversion that the margin text provides, being generally interesting but not essential to the understanding of the text. Of course, on those pages without the margin comments, approximately one‐third of the page is blank. The figures, including the digital radiography images, are of good quality.

The text is written at approximately the college freshman level with almost no calculus. Using the book as a graduate‐level medical physics text would require considerable supplementing with outside material. However, this may prove to be worthwhile because this text provides solid background material.

The authors may well have struggled with the problem of history versus currency. For my taste, history seemed to win too frequently. For example, Chapter 8, Treatment Planning by Manual Methods, contains methods that are of little use today. The useful content of this chapter could have been easily dispersed into other chapters. In Chapter 11, there was relatively too much discussion of obsolete treatment‐planning techniques and not enough discussion of modern algorithms that are more directly related to physics. In Chapter 12, it was not made clear that radium‐226 is no longer a source authorized for medical use. Generally, the book provides too little information on modern techniques while overemphasizing outmoded ones. In the final chapter, Advances in Radiation Therapy, only a single reference is included from the last decade, although more recent advances, such as intensity‐modulated radiotherapy, are discussed in other chapters.

It is my opinion that this book is best suited for a college‐level (interdisciplinary) medical physics course or as a text for radiation therapists. The historical background does give perspective to modern radiation oncology physics. The breadth is impressive, and students would have every opportunity to meet the stated objective of attaining “a solid foundation in the physics of radiation therapy.”

